# Sphingosine-1-phosphate induces pro-remodelling response in airway smooth muscle cells

**DOI:** 10.1111/all.12489

**Published:** 2014-09-06

**Authors:** E Fuerst, H R Foster, J P T Ward, C J Corrigan, D J Cousins, G Woszczek

**Affiliations:** 1 Division of Asthma, Allergy and Lung Biology, King's College LondonLondon, UK; 2 MRC & Asthma UK Centre in Allergic Mechanisms of AsthmaLondon, UK; 3 Department of Infection, Immunity and Inflammation, University of LeicesterLeicester, UK

**Keywords:** airway smooth muscle, asthma, remodelling, sphingosine-1-phosphate

## Abstract

**Background:**

Increased proliferation of airway smooth muscle (ASM) cells leading to hyperplasia and increased ASM mass is one of the most characteristic features of airway remodelling in asthma. A bioactive lipid, sphingosine-1-phosphate (S1P), has been suggested to affect airway remodelling by stimulation of human ASM cell proliferation.

**Objective:**

To investigate the effect of S1P on signalling and regulation of gene expression in ASM cells from healthy and asthmatic individuals.

**Methods:**

Airway smooth muscle cells grown from bronchial biopsies of healthy and asthmatic individuals were exposed to S1P. Gene expression was analysed using microarray, real-time PCR and Western blotting. Receptor signalling and function were determined by mRNA knockdown and intracellular calcium mobilization experiments.

**Results:**

S1P potently regulated the expression of more than 80 genes in human ASM cells, including several genes known to be involved in the regulation of cell proliferation and airway remodelling (HBEGF, TGFB3, TXNIP, PLAUR, SERPINE1, RGS4). S1P acting through S1P_2_ and S1P_3_ receptors activated intracellular calcium mobilization and extracellular signal-regulated and Rho-associated kinases to regulate gene expression. S1P-induced responses were not inhibited by corticosteroids and did not differ significantly between ASM cells from healthy and asthmatic individuals.

**Conclusion:**

S1P induces a steroid-resistant, pro-remodelling pathway in ASM cells. Targeting S1P or its receptors could be a novel treatment strategy for inhibiting airway remodelling in asthma.

Airway remodelling in asthma is characterized by increased airway smooth muscle (ASM) mass, epithelial and goblet cell hyperplasia, angiogenesis and reticular basement membrane thickening caused by a repeated process of injury and repair (1). Concomitant bronchial mucosal inflammation has been cited as one possible cause of remodelling (2), but the lack of efficacy of corticosteroids in inhibiting remodelling suggests that other pathogenic pathways are also involved (3). Indeed, simple compressive stress resulting from bronchoconstriction has been demonstrated to trigger a pro-fibrotic response reminiscent of remodelling in the absence of eosinophilic inflammation (4). This mechanotransduction pathway has been linked with growth factor shedding and heparin-binding epidermal growth factor-like growth factor (HB-EGF) signalling (5).

Sphingosine-1-phosphate (S1P) is a bioactive sphingolipid involved in mediating diverse cellular processes including cell growth, proliferation, survival and migration (6). S1P plays an important role in lymphocyte migration (7), but accumulating evidence implicates the involvement of S1P in pro-fibrotic and pro-remodelling responses in the lungs. Concentrations of S1P were increased in patients with idiopathic pulmonary fibrosis (IPF) to a degree which correlated with lung function and aberrant epithelial to mesenchymal transition (EMT) (8). S1P has also been found to promote the growth of fibroblasts and production of extracellular matrix proteins (9, 10). All of these processes have been associated with fibrotic lung remodelling.

In the case of asthma, genetic and functional studies have strongly linked genes in the sphingolipid pathway, in which S1P is the most prominent mediator, with the risk of developing the disease (11–13). Increased concentrations of S1P were also found in the bronchoalveolar lavage (BAL) fluid of asthmatics, but not healthy individuals following allergen challenge (14), and S1P has been shown to be involved in the regulation of allergic inflammation and bronchial hyper-responsiveness in murine ‘models’ of the disease (15, 16).

Airway smooth muscle hyperplasia, driven at least partly by mediators such as transforming growth factor β (TGF-β) and HB-EGF, is considered to be a major contributor to airway remodelling, obstruction and hyper-responsiveness in asthma (17–19). Nevertheless, the capacity for S1P to act directly on human ASM cells and contribute to remodelling has not been extensively studied. One study showed that S1P significantly increased proliferation of human ASM cells and stimulated IL-6 secretion (14). It has also been shown that S1P may regulate ASM contractility *in vitro* (20), but *in vivo* evidence in humans is lacking. The best–characterized activities of S1P have been attributed to signalling through a family of specific G-protein coupled receptor (GPCRs), named S1P_1–5_ (21). As the role of S1P and its receptors in human ASM functions and asthma remodelling has not been well understood, we aimed to investigate the effect of S1P on signalling and regulation of gene expression in ASM cells from healthy and asthmatic individuals.

## Materials and methods

### Patients

Airway smooth muscle cells from healthy and asthmatic individuals were obtained by deep endobronchial biopsy at fibreoptic bronchoscopy with the approval of the Research Ethics Committees of Guy's Hospital (10/H0804/66). Samples were obtained from 13 healthy volunteers (8F, 5M) and five asthmatic patients (2F, 3M) (three mild and two moderate asthmatics defined according to GINA guidelines and characterized in Data S1).

### Cell culture

Airway smooth muscle cells were grown from bronchial biopsies by explant culture as previously described (22, 23) and characterized as described in Data S1.

### Microarray analysis

Total RNA was isolated, processed and hybridized to the Affymetrix Human Exon 1.0 ST (Affymetrix, Cleveland, OH, USA) and analysed using the Partek Genomics Suite (Partek, St Louis, MO, USA) as described in Data S1. Data were submitted to Gene Expression Omnibus database (accession number GSE58657).

### Real-time PCR

Expression of mRNA encoding selected genes was measured using real-time PCR, ABI Prism 7900 (Applied Biosystems, Life Technologies, Paisley, UK) as described in Data S1.

### Western blot analysis

Total protein lysates or membrane proteins were isolated as described in Data S1 and proteins detected using primary antibodies against PTGS2 (COX2) (Clone CX229; Cayman Chemical, Ann Arbor, MI, USA), HBEGF (BioAcademia, Osaka, Japan), TXNIP (Clone JY1; Medical & Biological Laboratories, Nagoya, Japan) and control GAPDH (Clone 6C5; GeneTex, Irvine, CO, USA).

### S1P_2_ and S1P_3_ knockdown

Human ASM cells were transfected as described in Data S1 with 10 nM Silencer Select Validated siRNA s4454 (Ambion, Life Technologies, Paisley, UK) and 20 nM 27mer siRNA SR306152A (Origene, Rockville, MD, USA) and respective negative controls using Lipofectamine 2000 (Life Technologies, Paisley, UK) for S1P_3_ and S1P_2_ knockdown, respectively.

### Calcium mobilization assay

Calcium mobilization assays were performed using the FLIPR calcium 4 assay kit (Molecular Devices, Wokingham, UK) as previously described (24) and presented in Data S1.

## Results

### S1P induces gene expression in human ASM cells

**Figure 1:**
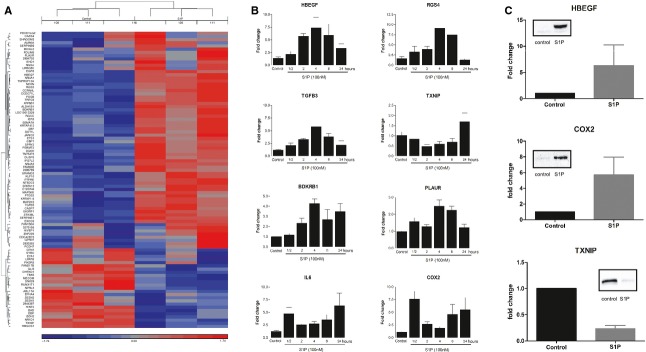
S1P regulates gene expression in human airway smooth muscle (ASM) cells. (A) ASM cells from three healthy donors were stimulated with S1P (100 nM) for 4 h, and gene expression was analysed using Affymetrix Exon 1.0 ST microarrays. Hierarchical clustering of significantly (anova *P* < 0.05, >2-fold change) regulated genes is presented as a heatmap. (B) Time course analysis of S1P-induced gene expression using real-time PCR. Data from three experiments (mean ± SEM) from different healthy donors are presented as a fold change in comparison with medium control cells. (C) Protein expression in response to S1P (100 nM). Cells were stimulated with S1P for 6 h for HBEGF (membrane proteins) and for 8 h for PTGS2 (COX2) and TXNIP, proteins extracted and analysed by Western blotting. Representative blots and densitometry data from three different donors presented as fold change (mean ± SEM) in relation to GAPDH expression.

Microarray analysis identified 88 genes regulated by S1P in ASM cells by twofold or more (Fig. 1A, Table S1), including genes involved in cell proliferation and airway remodelling (HBEGF, TGFB3, TXNIP, PLAUR, SERPINE1), intracellular signalling (RGS4, RGS2, DUSP5, MAP2K3, DGKH) and regulation of transcription (NR4A1, NR4A3, EGR3, FOSB). To further investigate S1P-induced effects, mRNA (Fig. 1B) and protein expression (Fig. 1C) of several significantly modified genes (HBEGF, RGS4, TGFB3, BDKRB1, PLAUR, TXNIP, PTGS2) were analysed over time by real-time PCR and Western blotting, confirming the microarray findings. Even though IL-6 has previously been shown to be induced by S1P in human ASM cells (14), it was not significantly upregulated by S1P in our microarray experiment. This is probably due to different regulation of transcription, with maximum increase observed after 24 h of S1P stimulation (Fig. 1B). Genes, most highly upregulated (HBEGF, RGS4) and downregulated (TXNIP) at 4 h, were selected for further analysis. S1P concentration of 100 nM was chosen for further experiments showing submaximal responses for calcium signalling and gene expression.

### S1P_2_ and S1P_3_ mediate gene regulation in human ASM cells

**Figure 2:**
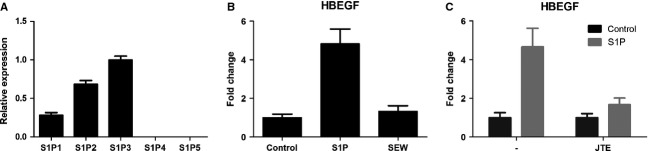
S1P signals through S1P receptors expressed in human airway smooth muscle (ASM) cells. (A) S1P receptor expression (mRNA) in ASM cells analysed by real-time PCR. Data from seven healthy controls are expressed relative to S1P_3_ expression and compared with internal 18S rRNA expression (mean ± SEM). (B) Cells were stimulated with S1P (100 nM), SEW2871 (SEW) (100 nM) or medium control for 4 h, and gene expression was analysed using real-time PCR. Data from three healthy donors are expressed as fold change relative to medium control (mean ± SEM). (C) Regulation of HBEGF expression by S1P_2_ inhibitor JTE-013 (JTE). Cells were pretreated with JTE-013 (1 μM), stimulated with S1P for 4 h and analysed using real-time PCR (*n* = 3, mean ± SEM).

**Figure 3:**
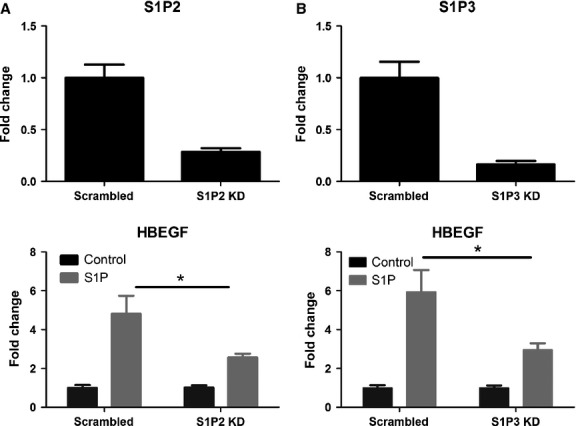
S1P signals through S1P_2_ and S1P_3_ in airway smooth muscle (ASM) cells. S1P_2_ (A) and S1P_3_ (B) mRNA expression was measured in un-stimulated cells transfected with scrambled siRNA and S1P_2_ (A) or S1P_3_ (B) siRNA (S1P2 KD/S1P3 KD) to confirm knockdown. HBEGF expression was measured after 4-h stimulation with S1P (100 nM) or medium control in control samples and S1P2 (A) or S1P3 (B) siRNA-treated cells. Data presented from three experiments from different healthy donors (mean ± SEM). *P < 0.05, one-way anova with Bonferroni post-test.

Real-time PCR identified S1P_2_ and S1P_3_ as being the most highly expressed, with lower expression of S1P_1_, and lack of S1P_4_ and S1P_5_ (Fig. 2A). SEW2871 (selective S1P_1_ agonist) did not modify gene expression (Fig. 2B), while JTE-013 (selective S1P_2_ antagonist) almost completely inhibited S1P-induced HBEGF expression (Fig. 2C). S1P_2_ downregulation using siRNA knockdown caused a significant reduction of the S1P-induced increase in HBEGF expression (Fig. 3A). As with S1P_2_ knockdown, S1P_3_ knockdown significantly reduced S1P-induced HBEGF mRNA expression compared with control cells (Fig. 3B). Similarly, S1P_2_ and S1P_3_ blockade or knockdown altered the expression of RGS4 and TXNIP (data not shown). Altogether, these data demonstrate that both S1P_2_ and S1P_3_ are involved in S1P-induced gene regulation in human ASM cells.

### S1P signals through intracellular calcium, Erk- and Rho-associated kinases for regulation of gene expression

**Figure 4:**
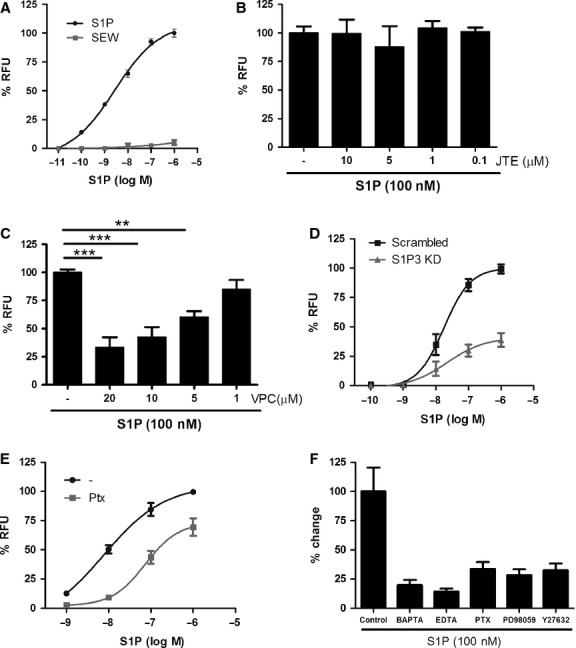
S1P signals through calcium, Erk and Rho-associated kinase activation. (A) Concentration response curve of calcium mobilization in response to S1P and SEW2871 (SEW). Cells were pretreated with different concentrations of (B) JTE-013 (JTE) or (C) VPC23019 (VPC) prior to stimulation with S1P (100 nM), and calcium mobilization was measured. **P < 0.01 and ***P < 0.001, one-way anova with Bonferroni post-test. (D) Calcium mobilization induced by S1P in S1P_3_ siRNA-treated cells and control cells. (E) Airway smooth muscle (ASM) cells were pretreated with pertussis toxin (Ptx) (100 ng/ml) for 18 h followed by stimulation with S1P, and calcium mobilization was measured. All data are expressed as percentage of maximum response to S1P (in relative fluorescence units – RFU) from three to four experiments with different healthy donors (mean ± SEM). (F) ASM cells were treated with BAPTA-AM for 30 min (9 μM) (BAPTA), EDTA (2.5 mM) for 5 min, pertussis toxin (PTX) (100 ng/ml) for 18 h, PD98059 (10 μM) for 10 min and Y27632 (1 μM) for 10 min prior to 4 h S1P (100 nM) stimulation. Regulation of HBEGF expression was analysed by real-time PCR, and data from three experiments from different healthy donors are presented as percentage of changes compared with S1P-stimulated samples (mean ± SEM).

To investigate downstream signalling of S1P_2_ and S1P_3_, we studied the effect of S1P on calcium mobilization in ASM cells. S1P induced a concentration dependent increase in intracellular calcium (EC_50_ = 2.7 × 10^−9^M) (Fig. 4A). The S1P_1_ agonist SEW2871 and the S1P_2_ antagonist JTE-013 did not affect calcium mobilization, excluding S1P_1_ and S1P_2_ as receptors signalling through calcium (Fig. 4 A,B). In contrast, pretreatment of ASM cells with VPC23019, a S1P_1_ and S1P_3_ antagonist, prior to S1P stimulation significantly reduced the release of intracellular calcium, suggesting that S1P_3_ is a receptor signalling through calcium in human ASM cells (Fig. 4C). Furthermore, S1P_3_ siRNA-treated cells showed reduced calcium mobilization in response to S1P (Fig. 4D) confirming this finding. S1P-induced calcium mobilization was mediated in part by G_i_ as pretreatment of ASM cells with pertussis toxin (PTX) partially inhibited S1P-induced calcium flux (Fig. 4E). To further analyse signalling pathways involved in S1P-induced gene regulation, inhibitors of calcium (BAPTA-AM and EDTA, intracellular and extracellular calcium chelators, respectively), PTX, inhibitors of extracellular signal-regulated kinase (Erk) and Rho-associated kinase pathways were used (Fig. 4F). All reagents inhibited the S1P-induced increase in HBEGF mRNA expression (Fig. 4F) and expression of other genes regulated by S1P (data not shown). These data are consistent with S1P signalling through calcium, Erk1/2 and ROCK to regulate gene expression in human ASM cells.

### S1P-induced gene expression is corticosteroid resistant

**Figure 5:**
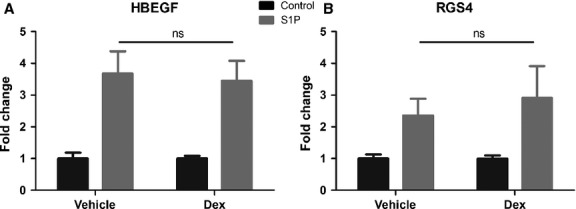
Corticosteroids do not inhibit S1P-induced gene expression. ASM cells were treated with dexamethasone (Dex) (100 nM) prior to 4-h stimulation with S1P (100 nM), and expression of (A) HBEGF and (B) RGS4 was measured using real-time PCR. Data from three different healthy donors are presented as fold change in comparison with controls (mean ± SEM). ns = nonstatistically significant; one-way anova with Bonferroni post-test.

As several genes regulated by S1P have been shown to be involved in airway remodelling, the effect of corticosteroids on S1P-induced gene expression was examined. ASM cells were pretreated with dexamethasone (Dex) and gene expression was measured following S1P stimulation. There was no significant difference between control and Dex-treated samples following S1P stimulation (Fig. 5), suggesting that corticosteroids do not target S1P-induced pathways in human ASM cells.

### Healthy and asthmatic ASM cells respond similarly to S1P stimulation

**Figure 6:**
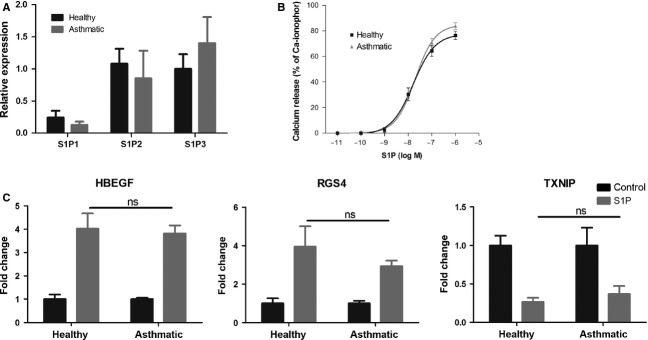
S1P signals similarly in healthy and asthmatic patients. (A) S1P receptor expression in airway smooth muscle (ASM) cells from healthy and asthmatic subjects analysed by real-time PCR. Data are expressed relative to 18S rRNA and normalized to S1P_3_ expression in healthy subjects (mean ± SEM). (B) ASM cells from healthy and asthmatic donors were stimulated with different concentrations of S1P, and calcium mobilization was measured. Data are expressed as percentage of the response to calcium ionophore (1 μM) (*n* = 3, mean ± SEM). (C) Cells from healthy and asthmatic donors were stimulated with S1P (100 nM) for 4 h, and gene expression was analysed using real-time PCR. Data are expressed as fold change (mean ± SEM). All data presented from experiments from three to four different healthy or asthmatic subjects. ns = nonstatistically significant; one-way anova with Bonferroni post-test.

To further characterize the role of S1P in inflammatory diseases such as asthma, ASM cells obtained from asthmatic patients were cultured and their responses to S1P were directly compared with ASM cells from healthy volunteers. There was no significant difference in mRNA expression of S1P receptors (Fig. 6A) or calcium mobilization induced by S1P (Fig. 6B) between ASM cells from healthy and asthmatic subjects (Fig. 6B). S1P increased HBEGF gene expression by more than 3.8-fold (Fig. 6C) in ASM cells from asthmatics, but the changes were not significantly different when compared to responses in ASM cells from healthy subjects. Similarly, there was no difference in the regulation of expression of other S1P-regulated genes, for example RGS4 and TXNIP (Fig. 6C) between healthy and asthmatics subjects.

## Discussion

Increased proliferation of ASM cells leading to hyperplasia and increased ASM mass is a characteristic feature of airway remodelling in asthma. We have identified a novel S1P-mediated pathway present in human ASM cells from healthy and asthmatic individuals, involved in regulation of pro-remodelling changes. S1P acting through specific S1P_2_ and S1P_3_ receptors potently regulated the expression of more than 80 genes in human ASM cells, including several genes known to be involved in the regulation of cell proliferation and airway remodelling (HBEGF, TGFB3, TXNIP, PLAUR, SERPINE1, RGS4). Interestingly, several of these genes (HBEGF, RGS4 and PLAUR) have previously been shown to be upregulated in airways of asthmatic individuals. HBEGF is believed to play a major role in driving remodelling changes in human airways. It has been reported to be expressed both in airway epithelium and in ASM cells of asthmatics and to be responsible for mediating cell proliferation through activation of EGF receptors (19, 25). Its expression also correlated with asthma severity. It has been suggested that HBEGF might be a biomarker for the active stage of ASM remodelling (19). In addition, HBEGF signalling through EGF receptors was identified as a main pathway activated by mechanical stress induced by airway constriction (5). Similarly, regulator of G-protein signalling 4 (RGS4) has been shown recently to be markedly upregulated in ASM cells from severe asthmatics and its expression correlated significantly with reduced pulmonary function (26). RGS4 was required for ASM hyperplasia and rendered cells poorly contractile, a feature characteristic of more severe, irreversible airway obstruction. Urokinase receptor (PLAUR, uPAR), also upregulated by S1P in ASM cells, was reported to be increased in patients with asthma and led to attenuated wound repair, a process contributing to development and progression of airway remodelling in asthma (27). Thioredoxin-interacting protein (TXNIP), also called vitamin D3 upregulated protein (VDUP1), was the gene most potently downregulated by S1P in our study. Interestingly, TXNIP has marked antiproliferative effects in smooth muscle cells acting through a suppression of the thioredoxin (TRX) system in response to reactive oxygen species (ROS) and mitogenic factors (28). It was suggested that TXNIP is a critical molecular switch in the transduction of pro-oxidant mitogenic signals upon platelet-derived growth factor (PDGF) and thrombin stimulations. Thus, S1P-mediated downregulation of TXNIP in ASM cells might be expected to have a pro-proliferative effect, acting in an additive or synergistic way with HBEGF or other mitogenic signals as reported previously for S1P and thrombin in ASM cells (14).

S1P binds with low nanomolar affinity to five GPCRs, S1P_1–5_. We found that three S1P receptors are expressed in ASM cells at the mRNA level, with S1P_2_ and S1P_3_ being the most predominant. We have also shown that S1P signals through S1P_2_ and S1P_3_ to regulate gene expression in ASM cells. Intracellular calcium mobilization and activation of Erk- and Rho-associated kinase pathways are required for transcriptional regulation. Our data suggest that S1P_3_, coupled to G_q_ and G_i_, is the major S1P receptor signalling through intracellular calcium mobilization and Erk activation in response to low nanomolar S1P concentrations. A similar signalling pathway for S1P_3_ has been described in lung epithelial cells leading to cytosolic phospholipase A2α activation and arachidonic acid synthesis (29). Nevertheless, S1P_2_ activation is also required for S1P-induced gene regulation as the selective S1P_2_ antagonist JTE-013 and S1P_2_ knockdown potently inhibited gene expression. In contrast to S1P_3_, S1P_2_ did not signal through calcium, but probably couples to G_12/13_ and activates Rho-associated kinase pathway in ASM cells. Based on our data, it can be suggested that both S1P receptors, S1P_2_ and S1P_3_, are needed for regulation of gene expression in ASM cells. These findings may be relevant for clinical applications, as it suggests that inhibition of S1P receptors could be potentially effective to prevent pro-remodelling changes.

S1P is a potent lipid mediator regulating diverse biological functions of many cell types, from proliferation and survival to migration and secretion (30). Constitutive concentrations of S1P in most tissues are very low, but high nanomolar concentrations can be found in blood and lymph, creating a sharp circulation-tissue S1P gradient responsible for trafficking of immune cells from secondary lymphoid tissues to blood vessels (21). Upon activation, many immune cells can release S1P, including mast cells, neutrophils, platelets and mononuclear cells. For example, activation of mast cells through the high affinity immunoglobulin E receptor (FcεRI) induces production and secretion of S1P, important for mast cell degranulation and for increased local tissue S1P concentrations (31). The observations that increased concentrations of S1P are found in BAL of asthmatic individuals following allergen challenge (14) and that infiltration of ASM by mast cells is associated with disordered airway function in asthma (32) suggest that S1P produced locally by activated mast cells may directly activate ASM cells. We have not observed significant differences in S1P signalling and regulation of gene expression between ASM cells from healthy and asthmatic subjects, but we cannot exclude possibility that this was due to small number of subjects studied, culture conditions or corticosteroid treatment (asthmatics) rather than lack of real *in vivo* differences. However, based on our data, we can speculate that it is repetitive exposure, rather than enhanced responsiveness, to S1P that determines increased expression of pro-remodelling factors observed in asthma, that is HBEGF, RGS4 and PLAUR. This is reminiscent of remodelling changes in human asthma proceeding in ‘waves’ during episodes of disease exacerbation (33). Interestingly, our observed effects of S1P were resistant to corticosteroid inhibition *in vitro*. Again, this is reminiscent of reports of remodelling changes in asthma being resistant to corticosteroid inhibition/reversal (34).

In summary, the growing perception that S1P is an important pro-inflammatory mediator in asthma and other inflammatory diseases (35–37), its ability to induce pro-fibrotic changes in lung fibroblasts (10) and our present findings that it may facilitate, through upregulation of HBEGF expression and other mechanisms, the mechanotransduction pathway caused by bronchoconstriction which results in remodelling all pinpoint S1P as a key molecular target in asthma, particularly in view of its potential insensitivity to corticosteroid inhibition.

## Supporting Information

Table S1 List of significantly regulated genes by S1P in ASM cells.
