# Supplementary material for: Sphingosine-1-phosphate induces pro-remodelling response in airway smooth muscle cells
Source: Allergy. 2014 Sep 6;69(11):1531–9. doi: 10.1111/all.12489 (PMC4329332; doi:10.1111/all.12489)
Supplement: Table S1 — List of significantly regulated genes by S1P in ASM cells. [file all0069-1531-sd1.docx]

Table S1. List of significantly regulated genes by S1P in ASM cells.

| Gene Symbol | Gene Name | Fold Change (S1P vs control) | *P* value |
| --- | --- | --- | --- |
| HBEGF | heparin-binding EGF-like growth factor | 9.19 | 0.0206 |
| GBP1 | guanylate binding protein 1, interferon-inducible | 7.71 | 0.0297 |
| NR4A1 | nuclear receptor subfamily 4, group A, member 1 | 7.64 | 0.0063 |
| NR4A3 | nuclear receptor subfamily 4, group A, member 3 | 7.31 | 0.0006 |
| DUSP5 | dual specificity phosphatase 5 | 6.33 | 0.0345 |
| LOC100133299 | GALI1870 | 5.93 | 0.0277 |
| RGS4 | regulator of G-protein signaling 4 | 5.55 | 0.0106 |
| EGR3 | early growth response 3 | 5.12 | 0.0361 |
| PTGS2 | prostaglandin-endoperoxide synthase 2 (COX2) | 3.95 | 0.0325 |
| BDKRB1 | bradykinin receptor B1 | 3.68 | 0.0277 |
| TGFB3 | transforming growth factor, beta 3 | 3.58 | 0.0033 |
| TNFAIP3 | tumor necrosis factor, alpha-induced protein 3 | 3.53 | 0.0154 |
| KLF10 | Kruppel-like factor 10 | 3.52 | 0.0042 |
| ETS1 | v-ets erythroblastosis virus E26 oncogene homolog 1 (avian) | 3.51 | 0.0208 |
| TRPC6 | transient receptor potential cation channel, subfamily C | 3.49 | 0.0005 |
| STK38L | serine/threonine kinase 38 like | 3.49 | 0.0060 |
| PTPRE | protein tyrosine phosphatase, receptor type, E | 3.13 | 0.0169 |
| CCDC71L | coiled-coil domain containing 71-like | 3.11 | 0.0199 |
| KRTAP1-5 | keratin associated protein 1-5 | 3.04 | 0.0244 |
| PDLIM5 | PDZ and LIM domain 5 | 3.02 | 0.0371 |
| JARID2 | jumonji, AT rich interactive domain 2 | 3.01 | 0.0056 |
| FAM65B | family with sequence similarity 65, member B | 2.90 | 0.0007 |
| HIVEP1 | human immunodeficiency virus type I enhancer binding protein | 2.88 | 0.0269 |
| TNFRSF12A | tumor necrosis factor receptor superfamily, member 12A | 2.86 | 0.0072 |
| RGS2 | regulator of G-protein signaling 2, 24kDa | 2.77 | 0.0183 |
| DOT1L | DOT1-like, histone H3 methyltransferase (S. cerevisiae) | 2.72 | 0.0055 |
| PDCD1LG2 | programmed cell death 1 ligand 2 | 2.71 | 0.0373 |
| MAP2K3 | mitogen-activated protein kinase kinase 3 | 2.71 | 0.0240 |
| SRF | serum response factor | 2.67 | 0.0049 |
| ALDH1B1 | aldehyde dehydrogenase 1 family, member B1 | 2.65 | 0.0237 |
| SERPINE1 | serpin peptidase inhibitor, clade E | 2.64 | 0.0369 |
| DGKH | diacylglycerol kinase, eta | 2.59 | 0.0069 |
| NT5DC3 | 5'-nucleotidase domain containing 3 | 2.53 | 0.0161 |
| EXOC6 | exocyst complex component 6 | 2.49 | 0.0165 |
| NEXN | nexilin (F actin binding protein) | 2.49 | 0.0155 |
| RGCC | regulator of cell cycle | 2.48 | 0.0461 |
| AURKA | aurora kinase A | 2.43 | 0.0091 |
| IER3 | immediate early response 3 | 2.43 | 0.0410 |
| LRRC8C | leucine rich repeat containing 8 family, member C | 2.40 | 0.0279 |
| CASS4 | Cas scaffolding protein family member 4 | 2.35 | 0.0086 |
| FOSB | FBJ murine osteosarcoma viral oncogene homolog B | 2.33 | 0.0097 |
| SEMA7A | semaphorin 7A, GPI membrane anchor | 2.33 | 0.0283 |
| ZNF295 | zinc finger protein 295 | 2.30 | 0.0292 |
| PLAUR | plasminogen activator, urokinase receptor | 2.30 | 0.0149 |
| IFI27L2 | interferon, alpha-inducible protein 27-like 2 | 2.24 | 0.0165 |
| CDC42EP3 | CDC42 effector protein (Rho GTPase binding) 3 | 2.23 | 0.0255 |
| FAM150A | family with sequence similarity 150, member A | 2.23 | 0.0268 |
| FERMT2 | fermitin family member 2 | 2.18 | 0.0060 |
| SERPINB9 | serpin peptidase inhibitor, clade B (ovalbumin) | 2.18 | 0.0415 |
| KRTAP4-12 | keratin associated protein 4-12 | 2.15 | 0.0120 |
| MICAL2 | microtubule associated monoxygenase | 2.15 | 0.0144 |
| SH3RF1 | SH3 domain containing ring finger 1 | 2.14 | 0.0075 |
| CASP7 | caspase 7, apoptosis-related cysteine peptidase | 2.11 | 0.0034 |
| GPR37 | G protein-coupled receptor 37 | 2.09 | 0.0078 |
| CCRN4L | CCR4 carbon catabolite repression 4-like (S. cerevisiae) | 2.09 | 0.0167 |
| GRAMD3 | GRAM domain containing 3 | 2.09 | 0.0003 |
| SPRY2 | sprouty homolog 2 (Drosophila) | 2.08 | 0.0058 |
| EHD1 | EH-domain containing 1 | 2.07 | 0.0137 |
| PCDH7 | protocadherin 7 | 2.07 | 0.0225 |
| SPATA13 | spermatogenesis associated 13 | 2.04 | 0.0215 |
| MAP3K8 | mitogen-activated protein kinase kinase kinase 8 | 2.03 | 0.0055 |
| C12orf44 | chromosome 12 open reading frame 44 | 2.01 | 0.0025 |
| SHROOM3 | shroom family member 3 | 2.01 | 0.0465 |
| ATP8B1 | ATPase, aminophospholipid transporter, class I, type 8B | 2.00 | 0.0170 |
| ARL17A | ADP-ribosylation factor-like 17A | -2.02 | 0.0388 |
| BAD | BCL2-associated agonist of cell death | -2.02 | 0.0309 |
| HMGCS1 | 3-hydroxy-3-methylglutaryl-CoA synthase 1 (soluble) | -2.02 | 0.0363 |
| SYBU | syntabulin (syntaxin-interacting) | -2.05 | 0.0467 |
| TBX5 | T-box 5 | -2.07 | 0.0118 |
| EYA1 | eyes absent homolog 1 (Drosophila) | -2.10 | 0.0470 |
| RUNX1T1 | runt-related transcription factor 1 | -2.13 | 0.0323 |
| HINT2 | histidine triad nucleotide binding protein 2 | -2.16 | 0.0355 |
| EPHA4 | EPH receptor A4 | -2.21 | 0.0217 |
| FAM217B | family with sequence similarity 217, member B | -2.25 | 0.0418 |
| GLI3 | GLI family zinc finger 3 | -2.44 | 0.0045 |
| NPRL3 | nitrogen permease regulator-like 3 (S. cerevisiae) | -2.46 | 0.0431 |
| DBP | D site of albumin promoter (albumin D-box) binding protein | -2.47 | 0.0335 |
| SESN2 | sestrin 2 | -2.49 | 0.0049 |
| DHTKD1 | dehydrogenase E1 and transketolase domain containing 1 | -2.58 | 0.0488 |
| SESN1 | sestrin 1 | -2.66 | 0.0244 |
| PIK3R3 | phosphoinositide-3-kinase, regulatory subunit 3 | -2.75 | 0.0438 |
| LPAR6 | lysophosphatidic acid receptor 6 | -2.79 | 0.0067 |
| SNX29 | sorting nexin 29 | -2.86 | 0.0054 |
| MECOM | MDS1 and EVI1 complex locus | -2.93 | 0.0103 |
| BDH2 | 3-hydroxybutyrate dehydrogenase, type 2 | -2.95 | 0.0369 |
| NR1D1 | nuclear receptor subfamily 1, group D, member 1 | -3.05 | 0.0100 |
| CPN1 | carboxypeptidase N, polypeptide 1 | -3.38 | 0.0493 |
| TXNIP | thioredoxin interacting protein | -6.41 | 0.0194 |
