# Supplementary material for: Sphingosine-1-phosphate induces pro-remodelling response in airway smooth muscle cells
Source: Allergy. 2014 Sep 6;69(11):1531–9. doi: 10.1111/all.12489 (PMC4329332; doi:10.1111/all.12489)
Supplement: Data S1 — Material and Methods. [file all0069-1531-sd2.docx]

Online Data Supplement

**Sphingosine-1-phosphate induces pro-remodelling response in airway smooth muscle cells**

Elisabeth Fuerst, Holly R. Foster, Jeremy P.T. Ward, Christopher J. Corrigan, David J. Cousins, Grzegorz Woszczek

**Material and Methods**

**Chemicals and Reagents**

Sphingosine-1-phosphate (D-*erythro*) (Enzo Life Sciences), JTE-013, SEW2871, (all Cayman Chemical), VPC23019 (Avanti Polar Lipids), EDTA (Ambion), PD98059 (Merck), BAPTA-AM, Pertussis Toxin, Y27632, calcium ionophore (A23187), and dexamethasone (all Sigma-Aldrich Dorset, UK) were obtained from the manufacturers.

**Patients**

Airway smooth muscle cells from healthy and asthmatic individuals were obtained by deep endobronchial biopsy at fibreoptic bronchoscopy with the approval of the Research Ethics Committees of Guy’s Hospital (10/H0804/66). All volunteers provided written informed consent prior to any procedure. Samples were obtained from 13 healthy volunteers (8F, 5M) and 5 asthmatic patients (2F, 3M) (3 mild and 2 moderate asthmatics defined according to GINA guidelines). Healthy subjects had mean predicted forced expiratory volume in 1s (FEV1) of 105 ± 15% (mean ± SD) and PC20 to methacholine of > 16 mg/ml, asthmatics had mean predicted FEV1 of 88 ± 13% (mean ± SD), PC20 to methacholine < 8 mg/ml (1.74 ± 1.6 mg/ml, mean ± SD), >12% reversibility in FEV1 to 400 μg inhaled salbutamol and were managed with stable dosages of inhaled corticosteroids (fluticasone propionate < 250 μg daily or equivalent).

**Cell Culture**

Airway smooth muscle cells were grown from bronchial biopsies by explant culture. Fluorescent immunocytochemistry was used routinely to confirm that near-confluent, FBS-deprived ASM cells stained (>95%) for smooth muscle specific α-actin, desmin, and calponin. Cells in passages 3-8 were used in experiments. Prior to all experiments, ASM cells were serum starved for 18 hours.

**Microarray Analysis**

Total cellular RNA was isolated using the miRNeasy mini kit (Qiagen, Crawley, United Kingdom), DNAase treated (Ambion) and further processed with the Ambion WT Expression Kit (Applied Biosystems) according to the manufacturers’ instructions. Second cycle cDNA was fragmented, stained, and hybridized to the Affymetrix Human Exon 1.0 ST Arrays at 45°C for 17 hours using the Gene Chip WT Terminal Labelling and Hybridization Kit (Affymetrix). GeneChip fluidics station 450 (Affymetrix) was used for processing of the chips and fluorescent signals were detected with the GeneChip scanner 3000. Images were analysed with the GeneChip operating software (Affymetrix). Further analysis was performed with the Partek Genomics Suite (Partek, St Louis, Mo). RMA processing and quantile normalisation was applied to GC and sequence corrected probe intensities, and after Median Polish gene level probeset summarisation, differentially expressed genes were identified using ANOVA with a *P* value of less than 0.05.

**Real time PCR**

Expression of mRNA encoding selected genes was measured using real time PCR on an ABI Prism 7900 Sequence Detection System (Applied Biosystems). Commercially available primer probe sets: BDKRB1 - Hs00664201_s1, TXNIP - Hs01006900_g1, 18S rRNA - 4319413E (Applied Biosystems, Paisley, UK) and individually designed assays using the Universal Probe Library (UPL) (Roche): PTGS2- probe 69, primers TGGGAAGCCTTCTCTAACCTC, TTGAATCAGGAAGCTGCTTTT; HBEGF - probe 55, primers TGGGGCTTCTCATGTTTAGG, CATGCCCAACTTCACTTTCTC;

IL6- probe 40, primers GATGAGTACAAAAGTCCTGATCCA, CTGCAGCCACTGGTTCTGT; PLAUR– probe 82, primers CTGCAAGGGGAACAGCAC, GCTTTGGTTTTTCGGTTCG; RGS4- probe 18, primers CGGCTTCTTGCTTGAGGA, GGGAAGAATTGTGTTCACAGG; S1PR1- probe 70, primers TGCAGAATCTACTCCTTGGTCA, GAAATGTTCTTGCGGAACG; S1PR2– probe 61, primers CCACTCGGCAATGTACCTGT, ACGCCTGCCAGTAGATCG; S1PR3- probe 69, primers CTTACGACGCCAACAAGAGG, AAGGCAATGAGCCAGCAC; S1PR4 – probe 19, primers TTTGCTGGGCTGGAACTG, AGAGGATGTAGCGCTTGGAG; S1PR5- probe 27, primers GAGTGGCTCCCAGCAGTC, GCTGAAGCTCCCATCAAGG; TGFB3– probe 38, AAGAAGCGGGCTTTGGAC, CGCACACAGCAGTTCTCC, were used. Relative gene expression was normalized to 18S rRNA and expressed as fold change. Data were analysed using SDS2.1 software (Applied Biosystems).

**Western Blot Analysis**

Human ASM cells were grown in 6 well plates (1x10^5^ cells per well) for 24 h and serum starved before S1P stimulation. Total protein lysates were prepared using lysis buffer containing 1mM protease inhibitor cocktail (Roche) or membrane proteins were isolated using the Mem-Per Eukaryotic Membrane Protein Extraction Kit (Thermo Scientific). Proteins (25µg) were loaded onto a 10% Tris-glycine gel (Invitrogen) and transferred onto a nitrocellulose membrane (Invitrogen). The membrane was incubated with primary antibodies against PTGS2 (COX2) (Clone CX229, Cayman Chemical), HBEGF (BioAcademia), TXNIP (Clone JY1, Medical&Biological Laboratories) and control GAPDH (Clone 6C5, GeneTex) overnight at 4°C, followed by secondary, horseradish peroxidase-conjugated antibody (goat anti mouse IgG (Southern Biotech)). Blots were developed using ECL2 Western Blot Substrate (Pierce) or ECL Prime Western Blot Detection Reagent (Amersham-GE Healthcare) and visualised on a Chemidoc MP System (BioRad). Data were analysed using Image Lab 4.1. software (BioRad).

**S1P_2_ and S1P_3_ knockdown**

Human ASM cells were plated in a 6 well plate (1x10^5^ cells per well). After 24 hours, cells were transfected with Lipofectamine2000 (Life Technologies) in serum free medium for 6 hours according to manufacturer’s instructions. 10nM Silencer Select Validated siRNA s4454 (Ambion) and 20nM 27mer siRNA SR306152A (Origene) and respective negative controls were used for S1P_3_ and S1P_2_ knockdown, respectively. Cells were then cultured in normal ASM medium for 24 hours and subsequently serum-starved for 18 hours prior to S1P stimulation. Knockdown of receptors was verified by real time PCR and functional assays were performed 48 hours after transfection.

**Calcium mobilisation assay**

Calcium mobilisation assays were performed using the FLIPR calcium 4 assay kit (Molecular Devices, Eugene, Ore) as previously described. Briefly, human ASM cells were resuspended at 10,000 cells/100µl in ASM medium, plated onto a black wall 96 well plate, incubated for 24 hours and then serum starved overnight, before incubation for 1 hour with FLIPR loading buffer. Fluorescence was measured in a FlexStation 3 Microplate Reader (Molecular Devices) at 37°C for a total of 120 seconds with 17 seconds baseline detection prior to ligand addition. Inhibitors were added 5 minutes and pertussis toxin 18 hours prior to addition of ligands. Vehicle controls were employed on each plate and background subtracted from the results. Results were analysed with SoftMax Pro Software (Molecular Devices).

**Statistical analysis**

Data were analysed by one way analysis of variance (ANOVA) with Bonferroni post test. Differences were considered significant when p < 0.05.
